# The epidemiology of trauma and post-traumatic stress disorder in a representative cohort of young people in England and Wales

**DOI:** 10.1016/S2215-0366(19)30031-8

**Published:** 2019-03

**Authors:** Stephanie J Lewis, Louise Arseneault, Avshalom Caspi, Helen L Fisher, Timothy Matthews, Terrie E Moffitt, Candice L Odgers, Daniel Stahl, Jia Ying Teng, Andrea Danese

**Affiliations:** a Department of Child and Adolescent Psychiatry, Institute of Psychiatry, Psychology and Neuroscience, King's College London, London, UK; b Social, Genetic and Developmental Psychiatry Centre, Institute of Psychiatry, Psychology and Neuroscience, King's College London, London, UK; c Department of Biostatistics, Institute of Psychiatry, Psychology and Neuroscience, King's College London, London, UK; d Department of Psychological Medicine, Yong Loo Lin School of Medicine, National University of Singapore, Singapore; e Department of Psychology and Neuroscience and Department of Psychiatry and Behavioral Sciences, Duke University, Durham, NC, USA; f Department of Psychology and Social Behavior, University of California, Irvine, CA, USA; g National and Specialist CAMHS Clinic for Trauma, Anxiety, and Depression, South London and Maudsley NHS Foundation Trust, London, UK

## Abstract

**Background:**

Despite the emphasis placed on childhood trauma in psychiatry, comparatively little is known about the epidemiology of trauma and trauma-related psychopathology in young people. We therefore aimed to evaluate the prevalence, clinical features, and risk factors associated with trauma exposure and post-traumatic stress disorder (PTSD) in young people.

**Methods:**

We carried out a comprehensive epidemiological study based on participants from the Environmental Risk Longitudinal Twin Study, a population-representative birth-cohort of 2232 children born in England and Wales in 1994–95. At the follow-up home visit at age 18 years, participants were assessed with structured interviews for trauma exposure, PTSD, other psychopathology, risk events, functional impairment, and service use. Risk factors for PTSD were measured prospectively over four previous assessments between age 5 and 12 years. The key outcomes were the prevalence, clinical features, and risk factors associated with trauma exposure and PTSD. We also derived and tested the internal validity of a PTSD risk calculator.

**Findings:**

We found that 642 (31·1%) of 2064 participants reported trauma exposure and 160 (7·8%) of 2063 experienced PTSD by age 18 years. Trauma-exposed participants had high rates of psychopathology (187 [29·2%] of 641 for major depressive episode, 146 [22·9%] of 638 for conduct disorder, and 102 [15·9%] of 641 for alcohol dependence), risk events (160 [25·0%] of 641 for self-harm, 53 [8·3%] of 640 for suicide attempt, and 42 [6·6%] of 640 for violent offence), and functional impairment. Participants with lifetime PTSD had even higher rates of psychopathology (87 [54·7%] of 159 for major depressive episode, 43 [27·0%] of 159 for conduct disorder, and 41 [25·6%] of 160 for alcohol dependence), risk events (78 [48·8%] of 160 for self-harm, 32 [20·1%] of 159 for suicide attempt, and 19 [11·9%] of 159 for violent offence), and functional impairment. However, only 33 (20·6%) of 160 participants with PTSD received help from mental health professionals. The PTSD risk calculator had an internally validated area under the receiver operating characteristic curve of 0·74, indicating adequate discrimination of trauma-exposed participants with and without PTSD, and internally validated calibration-in-the-large of −0·10 and calibration slope of 0·90, indicating adequate calibration.

**Interpretation:**

Trauma exposure and PTSD are associated with complex psychiatric presentations, high risk, and significant impairment in young people. Improved screening, reduced barriers to care provision, and comprehensive clinical assessment are needed to ensure that trauma-exposed young people and those with PTSD receive appropriate treatment.

**Funding:**

The Medical Research Council, the National Institute of Child Health and Development, the Jacobs Foundation, the Nuffield Foundation, the National Society for Prevention of Cruelty to Children, the Economic and Social Research Council, the National Institute for Health Research, MQ, and Canadian Institutes for Advanced Research.

## Introduction

Traumas—namely, events that involve danger of death, injury, or sexual violation^1^—pose substantial challenges in clinical practice and public health, including the assessment and treatment of psychopathology in trauma-exposed individuals and planning of service provision.^2^, ^3^ These challenges are particularly salient in young people, who are exposed to the highest rates of trauma^4^, ^5^ and might be more vulnerable to the effects of stressors due to ongoing neurobiological, emotional, and social development.^6^ To address these challenges, it is necessary to characterise comprehensively the prevalence, clinical features, and risk factors associated with trauma exposure and subsequent post-traumatic stress disorder (PTSD) in young people. The existing literature offers key insights but also highlights major gaps in knowledge.

First, trauma exposure and PTSD in young people are prevalent: 15–82·5% of young people experience a traumatic event,^5^, ^7^, ^8^, ^9^, ^10^, ^11^, ^12^, ^13^ and 1·3–8·1% experience PTSD in their lifetime^5^, ^7^, ^10^, ^12^, ^13^, ^14^ (0·6–3·9% experience PTSD in a 12-month period),^13^, ^15^, ^16^ with estimates varying according to the assessment methods used, the range of events assessed, and the sampling frame. However, these estimates are largely obtained from studies undertaken in the USA more than a decade ago with now-obsolete diagnostic criteria (ie, DSM-III and DSM-IV). Therefore, examination of the prevalence of trauma exposure and PTSD in young people using the current DSM-5 criteria in a contemporary, non-USA sample is necessary to inform service planning.

**Research in context**

**Evidence before this study**

Relatively little is known about the epidemiology of trauma exposure and post-traumatic stress disorder (PTSD) in representative samples of young people. We searched PubMed up to July 1, 2018, with the following terms: (“post-traumatic stress disorder” OR “posttraumatic stress disorder” OR “post traumatic stress disorder” OR “PTSD”) AND (“young” OR “youth*” OR “adolescent*” OR “child*” OR “pediatric” OR “paediatric” OR “juvenile*”). We did not apply any date or language restrictions. We identified population-based studies of trauma exposure and PTSD in young people done in high-income countries. This search was supplemented by reviewing reference lists and forward citations of relevant articles. We identified 11 original studies. Taken together, these studies suggest that trauma exposure and PTSD in young people are prevalent, are associated with a large health burden, and are linked to pre-existing risk factors. However, there are several gaps in knowledge. First, these studies were largely done more than a decade ago in the USA (seven studies), UK (two studies), Germany (one study), and Switzerland (one study) based on now-obsolete diagnostic criteria for PTSD. The UK studies—the 1999 and 2004 British Child and Adolescent Mental Health Surveys—only estimated the point prevalence of PTSD. Second, the breadth of the investigations was often limited: only three studies assessed PTSD comorbidity; only two studies explored risk events and impairment; and none measured service use. Finally, studies to date have not tested the prediction performance of identified risk factors for PTSD to inform the development of screening programmes.

**Added value of this study**

To our knowledge, this study is the first comprehensive epidemiological study of trauma exposure and PTSD in young British people. We found that nearly one in three young people experienced trauma and one in four of those exposed to trauma developed PTSD by age 18 years. Trauma-exposed young people, and particularly those with PTSD, had complex psychiatric presentations, high risk of harm to themselves, and functional impairment. However, only a minority received help from health professionals. We provided initial, proof-of-principle evidence based on internal validation that psychosocial and clinical risk factors might be used to make individualised risk predictions with adequate prediction performance.

**Implications of all the available evidence**

Clinicians should be aware that young people exposed to trauma and those with PTSD typically have complex presentations, high risk, and significant impairment. Building expertise in assessment and treatment of trauma-related psychopathology could therefore be cost-saving for service providers. Further research is needed to better characterise the mechanisms underlying the link between trauma and psychopathology, to improve screening practices, and to reduce barriers to care.

Second, trauma exposure and PTSD in young people are associated with a large health burden. Previous research has shown that both trauma exposure and PTSD are associated with elevated risk of psychiatric disorders, suicidality, and functional impairment.^8^, ^10^, ^13^, ^17^, ^18^ However, some of these studies focused on subthreshold PTSD categories to compensate for the relatively low numbers of participants meeting full criteria for PTSD in these samples, and most of these studies examined a narrow range of clinical features. Therefore, more comprehensive clinical assessment in large samples might be necessary to capture fully the health burden linked to trauma and PTSD.

Finally, not all trauma-exposed young people develop PTSD, and identifying those at greatest risk is important for care provision. Previous research has found that the risk of PTSD after trauma exposure is greater in girls, children who experienced previous victimisation, who have a history of psychopathology, who lived in disadvantaged or disrupted families, or who were exposed to interpersonal types of index trauma.^5^, ^7^, ^8^, ^9^, ^10^, ^11^, ^12^, ^13^, ^17^, ^19^, ^20^ However, it is unclear whether these risk factors can be used clinically to provide individualised risk prediction.^21^ Therefore, evaluation of the accuracy of PTSD risk prediction is necessary to inform screening practices.

In this study, we aimed to address these knowledge gaps by assessing the prevalence, clinical features, and risk factors associated with trauma exposure and PTSD in young people. On the basis of the aforementioned evidence, we hypothesised that trauma and PTSD are prevalent and are associated with a large health burden, and that PTSD can be accurately predicted by established risk factors.

## MethodsStudy design and sample

We carried out a comprehensive epidemiological study based on participants from the Environmental Risk (E-Risk) Longitudinal Twin Study, a population-representative birth cohort of 2232 children born in England and Wales in 1994–95. The sample was drawn from a larger birth register of twins born in England and Wales in 1994–95.^22^ Full details about the sample are reported elsewhere.^23^ Briefly, the E-Risk sample was constructed in 1999–2000, when 1116 families (93% of those eligible) with same-sex 5-year-old twins participated in home-visit assessments. This sample comprised 56% monozygotic and 44% dizygotic twin pairs; sex was evenly distributed within zygosity (49% were male). Families were recruited to represent the UK population of families with newborn babies in the 1990s, on the basis of residential location throughout England and Wales and mother's age. Teenaged mothers with twins were over-selected to replace high-risk families who were selectively lost to the register through non-response. Older mothers having twins via assisted reproduction were under-selected to avoid an excess of well educated older mothers.

At follow-up, the study sample represented the full range of socioeconomic conditions in the UK, as reflected in the families' distribution on a neighbourhood-level socioeconomic index (ACORN [A Classification of Residential Neighbourhoods], developed by CACI Incorporation for commercial use in Great Britain)^24^: 25·6% of E-Risk families live in wealthy achiever neighbourhoods versus 25·3% nationwide, 5·3% in urban prosperity neighbourhoods versus 11·6%, 29·6% in comfortably off neighbourhoods versus 26·9%, 13·4% in moderate means neighbourhoods versus 13·9%, and 26·1% in hard-pressed neighbourhoods versus 20·7%. E-Risk under-represents urban prosperity neighbourhoods because such households are likely to be childless.

Follow-up home visits were done when the children were aged 7 years (98% participation), 10 years (96%), and 12 years (96%); and in 2012–14 at 18 years (93%). Home visits at ages 5, 7, 10, and 12 years included assessments with participants and their mother (or primary caretaker); the home visit at age 18 years included interviews only with participants. Each twin participant was assessed by a different interviewer. 2066 children participated in the E-Risk assessments at age 18 years, and the proportions of monozygotic (55%) and male same-sex (47%) twins were almost identical to those found in the original sample at age 5 years. The average age of the twins at the time of assessment was 18·4 years (SD 0·36); all interviews were done after the 18th birthday. Further details on characteristics of the sample at age 18 years are provided in the appendix (p 7). There were no differences between those who did and did not take part at age 18 years in terms of socioeconomic status assessed when the cohort was initially defined (χ^2^=0·86, p=0·65), intelligence quotient (IQ) scores at age 5 years (*t*=0·98, p=0·33), or internalising (*t*=0·40, p=0·69) or externalising (*t*=0·41, p=0·68) problems at age 5 years.

The Joint South London and Maudsley and the Institute of Psychiatry Research Ethics Committee approved each phase of the study. Parents gave informed consent and twins gave assent between 5 years and 12 years of age and then informed consent at age 18 years.

### Trauma exposure and PTSD diagnosis

Trauma exposure and PTSD were assessed at age 18 years using private structured interviews to ascertain DSM-5 diagnostic criteria.^1^ The assessment began with a question to identify participants who had been exposed to trauma during their lifetime (DSM-5 PTSD criterion A). Participants who reported trauma exposure were asked subsequent questions relating to the trauma they felt had affected them most, the index trauma.

Index traumas were classified to indicate mutually exclusive categories of traumas experienced, based on qualitative information gathered as part of the PTSD interview and the Juvenile Victimization Questionnaire 2nd Revision^25^ adapted as a clinical interview. The trauma dossiers were independently coded by two psychiatrists (inter-rater reliability of κ=0·89; appendix p 4).

Trauma-exposed participants were asked about symptoms to assess DSM-5 PTSD criteria of re-experiencing (criterion B), avoidance (criterion C), negative alterations in cognitions and mood (criterion D), and arousal (criterion E), since age 12 years. Participants who met the (B, C, D, E) symptom criteria for PTSD were asked how long their symptoms lasted (>1 month=criterion F). Participants were then asked whether they had symptoms in the past year for more than a month, and the degree to which these symptoms interfered with daily life in the past year on a scale from 1 (very little) to 5 (very much; ≥2=criterion G). Participants received a diagnosis of lifetime DSM-5 PTSD if they met criteria A–F, and a diagnosis of 12-month DSM-5 PTSD if they met criteria A–G and reported having symptoms in the past year for more than a month. We focused on lifetime PTSD diagnosis. However, we report results for 12-month PTSD diagnosis where relevant.

### Clinical features

At age 18 years, we assessed clinical features described in the appendix (pp 4, 5), including mental health conditions (major depressive episode, generalised anxiety disorder, psychotic symptoms, attention-deficit hyperactivity disorder, conduct disorder, alcohol dependence, cannabis dependence, other drug dependence, nicotine dependence), risk events (to self: self-harm, suicide attempt; to others: violent offence), functional impairment (not in education, employment, or training; social isolation; loneliness), and service use for mental health (general practitioner; psychologist, psychotherapist, or counsellor; psychiatrist).

### Risk factors

Between age 5 and 12 years, we prospectively assessed childhood characteristics described in the appendix (pp 5, 6), including individual characteristics (female sex, minority ethnicity, IQ, internalising symptoms, externalising symptoms, psychotic symptoms, victimisation, serious accident) and family characteristics (socioeconomic disadvantage, fewer than two biological parents at home, family history of mental illness). These characteristics were selected to comprehensively capture risk factors for PTSD after trauma exposure identified in previous studies.^5^, ^7^, ^8^, ^9^, ^10^, ^11^, ^12^, ^13^, ^17^, ^19^, ^20^, ^26^, ^27^, ^28^, ^29^

### Statistical analysis

We did analyses on three key outcomes: trauma exposure in the overall sample, to investigate the burden of trauma in the general population; PTSD in the overall sample, to investigate the burden of PTSD in the general population; and PTSD in trauma-exposed participants, to investigate the burden of PTSD among those exposed to trauma. First, to provide contemporary prevalence estimates based on DSM-5, we described the distribution of each of these outcomes. Second, to characterise the health correlates of trauma exposure and PTSD, we tested the association between each of these outcomes of interest (independent variable) and clinical features (dependent variables) using bivariate logistic regression analyses with robust standard errors accounting for clustering of twins within families. We undertook a sensitivity analysis to test whether the risk and impairment observed in young people exposed to trauma or with PTSD were explained by associated psychopathology, using multivariate analyses adjusted for other mental health conditions. Finally, to examine risk prediction performance, we tested whether selected childhood risk factors (independent variables) predicted PTSD in trauma-exposed young people (dependent variable) using bivariate and multivariate logistic regression analyses with robust standard errors, and we derived and tested the internal validity of a PTSD risk calculator based on the multivariate logistic regression model. To test the internal validity of this model, we used 1000 bootstrap resamples to obtain overfitting (optimism) bias-corrected estimates of prediction performance. To generate each bootstrap resample, a sample was randomly selected from the original trauma-exposed sample (n=605 trauma-exposed participants with complete data) with replacement. For each bootstrap resample, the model was trained on the bootstrap and then tested in the original trauma-exposed sample. The average train-test difference was used as an estimate of overfitting and adjusted for in the estimates of prediction performance.^30^ Model prediction performance was measured in terms of discrimination, calibration, and overall prediction performance. Discrimination was assessed with area under the receiver operating characteristic curve (AUC) analysis, which indicates the probability that a participant who developed PTSD had a higher model-based predicted risk of PTSD than a trauma-exposed participant who did not develop PTSD (perfect discrimination=1, no discrimination=0·5); calibration was assessed with calibration-in-the-large, which indicates the intercept of the calibration plot (perfect=0), and calibration slope (perfect=1); and overall prediction performance was assessed with the Brier score, which indicates the mean squared difference between observed and predicted probabilities of PTSD (perfect prediction=0; appendix pp 1, 2).

Where data were missing, in analyses of prevalence and clinical features, we used pairwise deletion, so that all available data were used; and in analyses of risk factors, we used listwise deletion, so that data from the same participants were used in bivariate and multivariate models enabling comparison.

We did the analyses using Stata (version 15), and R (version 3.4.2) including the rms package.

### Role of the funding source

The funders of the study had no role in study design, data collection, data analysis, data interpretation, or writing of the report. The corresponding author had full access to all the data in the study and had final responsibility for the decision to submit for publication.

## Results

**Figure 1:**
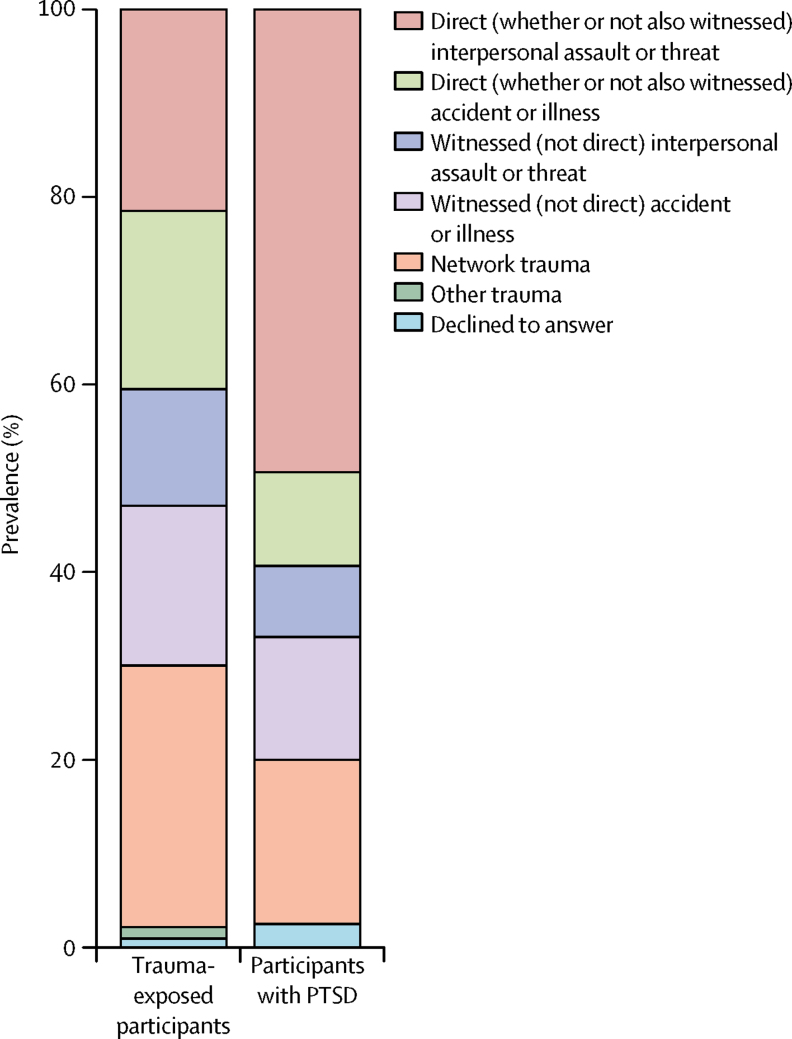
Prevalence of index trauma categories in trauma-exposed participants and in participants with PTSD The index traumas categorised as interpersonal assault or threat involved actions of another person intentionally causing or threatening death, injury, or sexual violation (eg, maltreatment by adults, bullying by peers), as opposed to index traumas categorised as accident or illness. Either trauma category might be directly experienced by the participant (ie, direct) or witnessed only (ie, witnessed). Other trauma categories were network trauma (ie, a traumatic event affecting someone in the participant's network that they learned details of, but did not directly experience or witness) or other trauma (ie, any other trauma that did not fall into the other categories). PTSD=post-traumatic stress disorder.

The lifetime prevalence of trauma exposure reported at age 18 years was 31·1% (642 of 2064). The median age at index trauma exposure was 15 years (IQR 13–17). The most common index trauma category was network trauma—a traumatic event affecting someone in the participant's network that they learned details of, but did not directly experience or witness—reported by 179 (27·9%) of 642 trauma-exposed participants. The next most common index trauma categories involved direct interpersonal assault or threat (138 [21·5%] of 642), and direct accident or illness (122 [19·0%] of 642; figure 1; appendix p 8).

The lifetime prevalence of PTSD by age 18 years in the overall sample was 7·8% (160 of 2063 [one participant did not answer questions about PTSD]), and the 12-month prevalence was 4·4% (90 of 2063). The lifetime prevalence of PTSD by age 18 years in trauma-exposed participants was 25·0% (160 of 641), and the 12-month prevalence was 14·0% (90 of 641).

The lifetime prevalence of PTSD was highest in participants who had experienced direct interpersonal assault or threat (79 [57·2%] of 138), particularly those who had been sexually assaulted (40 [74·1%] of 54) or physically assaulted (36 [61·0%] of 59; appendix p 8). Direct interpersonal assault or threat was also the index trauma reported by most participants with lifetime PTSD (79 [49·4%] of 160; figure 1; appendix p 8).

**Figure 2:**
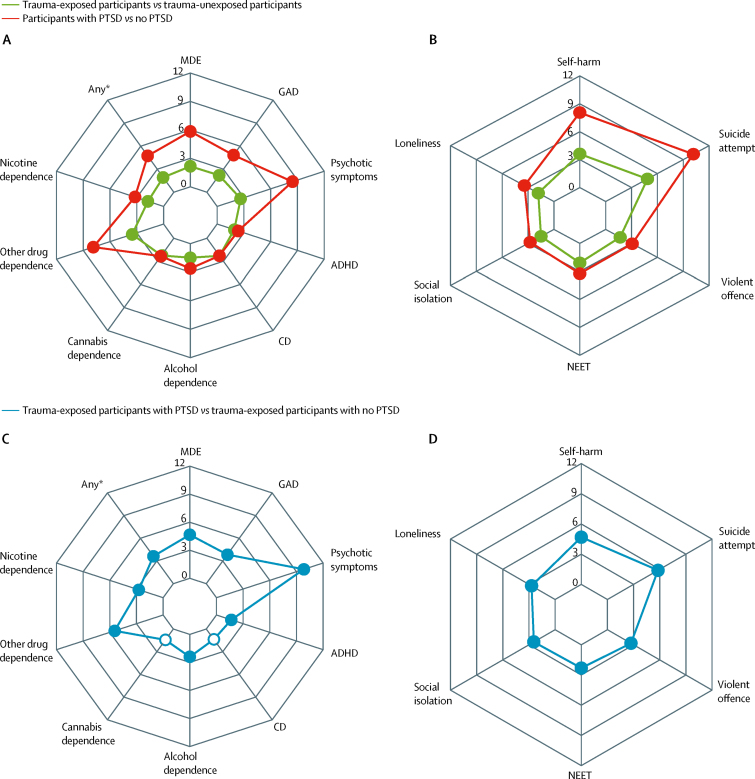
Risk of clinical features in the overall sample (n=2066) and the trauma-exposed participants (n=642) (A) Unadjusted ORs in the overall sample for mental health conditions. (B) Unadjusted ORs in the overall sample for risk events and functional impairment. (C) Unadjusted ORs in the trauma-exposed participants for mental health conditions. (D) Unadjusted ORs in the trauma-exposed participants for risk events and functional impairment. Filled circles signify significance in which p<0·05. Unfilled circles signify no significance in which p≥0·05. These results are detailed in the appendix (p 9). ADHD=attention-deficit hyperactivity disorder. CD=conduct disorder. GAD=generalised anxiety disorder. MDE=major depressive episode. NEET=not in education, employment, or training. ORs=odds ratios. PTSD=post-traumatic stress disorder. *Any mental health condition listed in (A) or (C).

Among trauma-exposed participants, the most common mental health conditions experienced in the 12 months before assessment were major depressive episode (187 [29·2%] of 641), conduct disorder (146 [22·9%] of 638), and alcohol dependence (102 [15·9%] of 641; table 1), which were also the most common conditions in trauma-unexposed participants. Trauma-exposed participants had higher rates of all nine measured mental health conditions than trauma-unexposed participants, and odds ratios (ORs) were highest for other drug dependence (3·52, 95% CI 1·36–9·12) and psychotic symptoms (2·64, 1·38–5·04; figure 2A; appendix p 9). Risk events were prevalent in trauma-exposed participants (160 [25·0%] of 641 for self-harm, 53 [8·3%] of 640 for suicide attempt, and 42 [6·6%] of 640 for violent offence; table 1), and were more common in trauma-exposed participants than in participants who had not experienced trauma (OR 3·61 [95% CI 2·77–4·70] for self-harm, 4·85 [2·89–8·13] for suicide attempt, and 1·68 [1·17–2·40] for violent offence; figure 2B; appendix p 9). Trauma-exposed participants also had higher rates of functional impairment and service use than trauma-unexposed participants (table 1; figure 2B; appendix p 9).

**Table 1:** Prevalence of clinical features

	**Overall sample (n=2066)**	**Trauma-unexposed participants (n=1422)**	**Trauma-exposed participants (n=642)**	**Overall sample with no PTSD (n=1903)**	**Trauma-exposed participants with no PTSD (n=481)**	**Trauma-exposed participants with PTSD (n=160)**
**Mental health conditions (past 12 months)**						
Major depressive episode	414/2063 (20·1%)	227/1420 (16·0%)	187/641 (29·2%)	326/1901 (17·1%)	99/481 (20·6%)	87/159 (54·7%)
Generalised anxiety disorder	153/2060 (7·4%)	79/1417 (5·6%)	74/641 (11·5%)	115/1897 (6·1%)	36/480 (7·5%)	38/160 (23·8%)
Psychotic symptoms	39/2063 (1·9%)	18/1420 (1·3%)	21/641 (3·3%)	23/1900 (1·2%)	5/480 (1·0%)	15/160 (9·4%)
Attention-deficit hyperactivity disorder	171/2061 (8·3%)	94/1418 (6·6%)	77/641 (12·0%)	144/1898 (7·6%)	50/480 (10·4%)	26/160 (16·3%)
Conduct disorder	309/2053 (15·1%)	162/1413 (11·5%)	146/638 (22·9%)	264/1891 (14·0%)	102/478 (21·3%)	43/159 (27·0%)
Alcohol dependence	263/2063 (12·7%)	161/1420 (11·3%)	102/641 (15·9%)	221/1900 (11·6%)	60/480 (12·5%)	41/160 (25·6%)
Cannabis dependence	89/2066 (4·3%)	45/1422 (3·2%)	44/642 (6·9%)	75/1903 (3·9%)	30/481 (6·2%)	14/160 (8·8%)
Other drug dependence	18/2066 (0·9%)	7/1422 (0·5%)	11/642 (1·7%)	11/1903 (0·6%)	4/481 (0·8%)	7/160 (4·4%)
Nicotine dependence	183/2062 (8·9%)	104/1419 (7·3%)	78/641 (12·2%)	147/1899 (7·7%)	43/480 (9·0%)	34/160 (21·3%)
Any of the above conditions	886/2038 (43·5%)	538/1402 (38·4%)	346/634 (54·6%)	762/1877 (40·6%)	224/475 (47·2%)	121/158 (76·6%)
**Risk events (since age 10–12 years)**						
Self-harm	280/2064 (13·6%)	120/1422 (8·4%)	160/641 (25·0%)	201/1902 (10·6%)	81/480 (16·9%)	78/160 (48·8%)
Suicide attempt	79/2063 (3·8%)	26/1422 (1·8%)	53/640 (8·3%)	46/1902 (2·4%)	20/480 (4·2%)	32/159 (20·1%)
Violent offence	99/2060 (4·8%)	57/1418 (4·0%)	42/640 (6·6%)	80/1898 (4·2%)	23/480 (4·8%)	19/159 (11·9%)
**Functional impairment (at time of assessment)**						
Not in education, employment, or training	239/2066 (11·6%)	128/1422 (9·0%)	110/642 (17·1%)	194/1903 (10·2%)	66/481 (13·7%)	43/160 (26·9%)
Social isolation	577/2061 (28·0%)	360/1417 (25·4%)	217/642 (33·8%)	497/1898 (26·2%)	137/481 (28·5%)	79/160 (49·4%)
Loneliness	541/2051 (26·4%)	320/1413 (22·6%)	221/636 (34·7%)	457/1889 (24·2%)	137/476 (28·8%)	83/159 (52·2%)
**Service use for mental health (past 12 months)**						
General practitioner	215/2064 (10·4%)	113/1421 (8·0%)	102/641 (15·9%)	166/1901 (8·7%)	53/480 (11·0%)	48/160 (30·0%)
Psychologist, psychotherapist, or counsellor	133/2065 (6·4%)	73/1421 (5·1%)	60/642 (9·3%)	102/1902 (5·4%)	29/481 (6·0%)	30/160 (18·8%)
Psychiatrist	45/2065 (2·2%)	21/1421 (1·5%)	24/642 (3·7%)	29/1902 (1·5%)	8/481 (1·7%)	16/160 (10·0%)

Data are n/N (%). Where data were missing, we have used pairwise deletion. A full description of these clinical features is provided in the appendix (pp 4, 5). PTSD=post-traumatic stress disorder.

Similarly, the most common mental health conditions experienced among participants with lifetime PTSD in the 12 months before assessment were major depressive episode (87 [54·7%] of 159), conduct disorder (43 [27·0%] of 159), and alcohol dependence (41 [25·6%] of 160; table 1). Participants with lifetime PTSD had higher rates of all nine measured mental health conditions than participants without PTSD in the overall sample, and ORs were highest for psychotic symptoms (8·44, 95% CI 4·41–16·15), other drug dependence (7·87, 3·00–20·62), major depressive episode (5·84, 4·21–8·10), and generalised anxiety disorder (4·83, 3·17–7·35; figure 2A; appendix p 9). Risk events were highly prevalent in participants with lifetime PTSD (78 [48·8%] of 160 for self-harm, 32 [20·1%] of 159 for suicide attempt, and 19 [11·9%] of 159 for violent offence; table 1), and were more common in participants with lifetime PTSD than in participants without PTSD in the overall sample (OR 8·05 [95% CI 5·64–11·48] for self-harm, 10·17 [6·21–16·65] for suicide attempt, and 3·08 [1·82–5·23] for violent offence; figure 2B; appendix p 9). Participants with lifetime PTSD also had greater functional impairment and service use than participants without PTSD (table 1; figure 2B; appendix p 9). Nevertheless, only a minority of participants with PTSD had accessed health services for mental health problems within the past year (48 [30·0%] of 160 from their general practitioner; 30 [18·8%] of 160 from a psychologist, psychotherapist, or counsellor; 16 [10·0%] of 160 from a psychiatrist; 33 [20·6%] from mental health professionals [overlap between groups]; table 1). Similar findings emerged when considering the 12-month PTSD diagnosis (appendix pp 10, 11).

Among trauma-exposed participants, those with lifetime PTSD had higher odds of all other mental health conditions (particularly psychotic symptoms [OR 9·83, 95% CI 3·72–25·97], other drug dependence [5·46, 1·58–18·84], major depressive episode [4·66, 3·22–6·76], and generalised anxiety disorder [3·84, 2·36–6·24]) than those without PTSD, except for conduct disorder and cannabis dependence (table 1; figure 2C; appendix p 9). Participants with lifetime PTSD had higher odds of risk events than trauma-exposed participants without PTSD (OR 4·69 [95% CI 3·16–6·95] for self-harm, 5·80 [3·18–10·56] for suicide attempt, and 2·70 [1·43–5·09] for violent offence). Participants with lifetime PTSD also had higher odds of functional impairment and service use than trauma-exposed participants without PTSD (table 1; figure 2D; appendix p 9). Similar findings emerged when considering the 12-month PTSD diagnosis (appendix pp 10, 11).

In multivariate analyses accounting for other mental health conditions, trauma-exposed participants or those with PTSD still had higher rates of self-harm, suicide attempts, and functional impairment than trauma-unexposed participants or those without PTSD (appendix p 12).

We next analysed risk factors for PTSD in trauma-exposed participants. In bivariate analyses, we found that girls and children with lower IQ, who had more internalising or psychotic symptoms, who experienced victimisation, who lived in more disadvantaged socioeconomic conditions, or who were exposed to direct interpersonal index traumas had greater risk of developing PTSD after trauma exposure (table 2). In multivariate analysis, childhood victimisation and direct interpersonal index trauma remained significant independent predictors of PTSD (table 2).

**Table 2:** Risk factors for PTSD in trauma-exposed participants

	Bivariate analyses*	**Multivariate analysis**†
**Individual characteristics**		
Female sex	1·97 (1·33–2·91)	1·51 (0·95–2·38)
Minority ethnicity	1·50 (0·66–3·43)	1·27 (0·61–2·38)
Child IQ‡	0·77 (0·63–0·94)	0·86 (0·68–1·09)
Child internalising symptoms‡	1·32 (1·04–1·57)	1·04 (0·82–1·33)
Child externalising symptoms‡	1·13 (0·96–1·33)	0·92 (0·71–1·21)
Child psychotic symptoms	2·15 (1·18–3·92)	1·56 (0·81–2·99)
Child victimisation	2·88 (1·97–4·20)	2·35 (1·49–3·70)
Child accident	1·16 (0·78–1·72)	1·19 (0·77–1·83)
**Family characteristics**		
Socioeconomic disadvantage	1·96 (1·33–2·89)	1·44 (0·92–2·23)
<2 biological parents at home	1·35 (0·92–1·98)	1·00 (0·63–1·58)
Family history of mental illness	0·83 (0·47–1·46)	0·79 (0·43–1·47)
**Index trauma category**		
Direct (whether or not also witnessed) interpersonal assault or threat	7·19 (4·68–11·06)	6·22 (3·96–9·75)

Data are OR (95% CI) for the associations between childhood characteristics and lifetime PTSD in trauma-exposed participants (n=605). PTSD=post-traumatic stress disorder. IQ=intelligence quotient. OR=odds ratio.

* Bivariate unadjusted associations.

† Multivariate associations, adjusted for the effects of all individual characteristics, family characteristics, and index trauma category. A full description of these childhood characteristics is provided in the appendix (pp 5, 6).

‡ Continuous variables were standardised; therefore, the OR relates to a 1 SD change.

**Figure 3:**
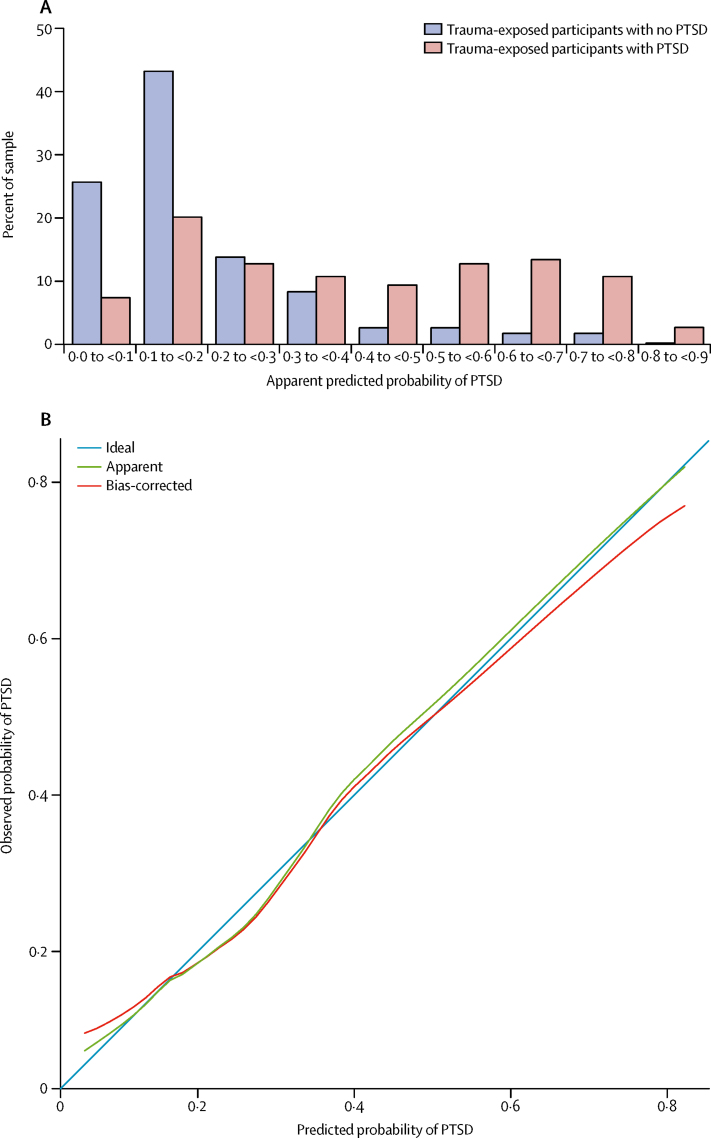
PTSD risk calculator prediction performance (A) Frequency distribution of predicted probabilities of PTSD in trauma-exposed participants without and with PTSD. (B) Calibration curve of the observed probabilities of lifetime PTSD in relation to the predicted probabilities of lifetime PTSD. The PTSD risk calculator was derived using the multivariate logistic regression model predicting lifetime PTSD in trauma-exposed participants. PTSD=post-traumatic stress disorder.

We developed a PTSD risk calculator based on this multivariate model. We found that the apparent predicted probabilities generated by the calculator were higher in participants with PTSD than in trauma-exposed participants without PTSD (figure 3A). We then tested the internal validity of the PTSD risk calculator. First, we assessed discrimination—ie, the ability of the model to distinguish between trauma-exposed participants who did or did not develop PTSD. We found that the internally validated AUC was 0·74, indicating adequate discrimination of trauma-exposed participants with and without PTSD. Second, we assessed calibration—ie, the degree of agreement between observed and model-based predicted risk of PTSD. We found that internally validated calibration-in-the-large was −0·10 and calibration slope was 0·90, indicating adequate calibration. The calibration curve showed that observed probabilities of PTSD were consistent with internally validated model predictions (figure 3B), particularly in the range 0·0–0·6, within which 95·2% (576 of 605) of the predictions fell (median 0·16, IQR 0·10–0·33). Finally, we assessed the overall prediction performance, which captures aspects of both discrimination and calibration. We found that this prediction model had an internally validated Brier score of 0·15, indicating adequate overall risk prediction performance. The risk calculator formula is provided in the appendix (p 3).

## Discussion

This contemporary population-representative cohort study provides new insights into the prevalence, clinical features, and risk factors associated with trauma and PTSD in young people. Our findings should be considered in the context of some limitations. First, we studied a cohort of twins, and results might not generalise to singletons. However, the prevalences of trauma, PTSD, and clinical features in this sample are broadly consistent with previous research of young people,^5^, ^7^, ^8^, ^9^, ^10^, ^11^, ^12^, ^13^, ^14^, ^15^, ^16^ supporting the generalisability of our findings. Second, PTSD was assessed based on DSM-5 criteria, which are broader than those used in the ICD-11 Revision.^31^ As such, the epidemiology of PTSD based on ICD-11 criteria will need further investigation. Third, the prevalence of risk factors and their contribution to PTSD risk might vary in different contexts. Therefore, the prediction model presented here needs to be validated in external samples before implementation in clinical practice or public health interventions (eg, post-disaster relief operations). Despite these limitations, our findings have implications for public health and clinical practice, and highlight new research directions.

With regard to prevalence, we found that 31·1% of young people experienced trauma and 7·8% developed PTSD by age 18 years. These results add to previous evidence,^4^, ^6^, ^7^, ^8^, ^9^, ^11^, ^12^, ^13^, ^15^ providing estimates using DSM-5 classification in a contemporary, European cohort. The results highlight the need to build clinical expertise to address the needs of young people who are exposed to trauma and develop PTSD. The risk of developing PTSD was greatest after direct interpersonal index traumas, such as maltreatment by adults or bullying by peers. Although only a fifth of trauma-exposed participants reported exposure to direct interpersonal trauma, this trauma category accounted for nearly half of PTSD cases. Therefore, our results highlight the important role of direct interpersonal trauma in PTSD burden among young people, and the need to better understand the origins of the risk associated with this trauma category.

With regard to clinical features, trauma-exposed young people were twice as likely as non-traumatised participants to develop a wide range of mental health conditions. PTSD was not the most common mental health condition in trauma-exposed young people as conditions with higher base rates, such as depression, conduct disorder, and alcohol dependence, were also the most prevalent conditions in this group. Furthermore, one in four trauma-exposed young people had self-harmed and one in 12 attempted suicide since age 12 years. Therefore, our results highlight the importance of assessing trauma-exposed young people for a wide range of mental health conditions including but not limited to PTSD. Assessment should also include careful consideration of the risk of suicide and self-harm.

Young people with PTSD were at high risk of other mental health problems: three in four had another mental health condition at age 18 years. Of note, PTSD was associated with additional clinical burden over and above trauma exposure, as young people with PTSD had higher odds of mental health conditions compared with trauma-exposed peers without PTSD. Therefore, our results suggest that young people with PTSD typically present with complex comorbid psychopathology, and thus require comprehensive psychiatric assessment and treatment. Clinicians should also be aware that co-occurring psychopathology can mask the diagnosis of PTSD in trauma-exposed young people.

Young people with PTSD also had high levels of risk and functional impairment, which were not explained by trauma exposure or other mental health conditions: half of young people with PTSD had self-harmed, one in five attempted suicide since age 12 years, one in four were not in education, employment, or training, and half experienced high social isolation or loneliness. Strikingly, despite their complex psychopathology, risk, and impairment, only one in three young people with PTSD had received help from their general practitioner for mental health problems within the past year, one in five saw a psychologist, psychotherapist, or counsellor, and one in ten saw a psychiatrist. Therefore, our results highlight the substantial unmet needs of young people with PTSD, and call for research to evaluate the effectiveness of screening for PTSD and to identify barriers to care provision for young people with PTSD.

With regard to PTSD risk prediction, our results provide initial, proof-of-principle evidence that assessment of psychosocial and clinical risk factors might be used to make individualised risk predictions with adequate performance using a PTSD risk calculator. Validation in external samples is essential as it could provide a clinically useful tool to identify trauma-exposed young people at greatest risk of developing PTSD.

In conclusion, young people exposed to trauma and those with PTSD typically have complex presentations. Building expertise in assessment and treatment of trauma-related psychopathology could therefore be cost-saving for service providers. Further research is needed to better characterise the mechanisms underlying the link between trauma and psychopathology, to improve screening practices, and to reduce barriers to care.
