# Supplementary material for: The epidemiology of trauma and post-traumatic stress disorder in a representative cohort of young people in England and Wales
Source: Lancet Psychiatry. 2019 Mar;6(3):247–56. doi: 10.1016/S2215-0366(19)30031-8 (PMC6384243; doi:10.1016/S2215-0366(19)30031-8)
Supplement: Supplementary appendix [file mmc1.pdf]

THE LANCET
Psychiatry

Supplementary appendix

This appendix formed part of the original submission and has been peer reviewed. We post it as supplied by the authors.

Supplement to: Lewis SJ, Arseneault L, Caspi A, et al. The epidemiology of trauma and post-traumatic stress disorder in a representative cohort of young people in England and Wales. *Lancet Psychiatry* 2019; **6:** 247–56.

Supplementary material for Lewis et al, The epidemiology of trauma and post-traumatic stress disorder in a representative cohort of young people in England and Wales

CONTENTS

Supplementary Methods .... Page 1

Supplementary Results .... Page 3

Supplementary Tables

- Table S1. Assessment of trauma-exposure, PTSD diagnosis, clinical features, and childhood risk factors .... Page 4
- Table S2. Characteristics of the sample .... Page 7
- Table S3. Prevalence of trauma exposure, PTSD, and PTSD burden, by index trauma type and category .... Page 8
- Table S4. Risk of clinical features .... Page 9
- Table S5. Prevalence of clinical features (12-month PTSD) .... Page 10
- Table S6. Risk of clinical features (12-month PTSD) .... Page 11
- Table S7. Risk of clinical features, adjusted for other mental health conditions .... Page 12
- Table S8. PTSD risk calculator sensitivity analysis to compare different methods of internal validation and penalized models .... Page 13
- Table S9. PTSD risk calculator sensitivity analysis to assess for bias in using twin data .... Page 14
- Table S10. PTSD risk score in trauma-exposed participants .... Page 15

Supplementary References .... Page 16

SUPPLEMENTARY METHODS

Analysis plan

The analysis plan was pre-registered with the E-Risk Study team, and is available online: https://sites.google.com/site/dunedineriskconceptpapers/e-risk-approved.

PTSD risk prediction internal validation

We derived a PTSD risk calculator based on a multivariate logistic regression model predicting PTSD in trauma-exposed participants (Table 2). We tested the internal validity of this model using 1,000 bootstrap resamples. To generate each bootstrap resample, a sample of n=605 was randomly selected from the original trauma-exposed sample (n=605 trauma-exposed participants with complete data) with replacement; that is, the probability of inclusion of any participant at each draw was independent of inclusion at previous draws, and therefore participants could be included in the bootstrap sample multiple times, once, or not at all. For each bootstrap sample, the model was trained on the bootstrap sample and then tested in the original trauma-exposed sample. The average train-test difference was used as an estimate of overfitting (optimism) and adjusted for in the estimates of prediction performance (Harrell's method).[1] Analyses were conducted using R 3.4.2[2] including the 'rms' package.[3] This method of internal validation was our preferred method because it has been found to perform as well as, or better than, other internal validation methods, and to provide stable prediction performance estimates with low bias.[4,5]

We also tested the internal validity of this logistic regression model using ten-fold cross-validation, for comparison. To generate ten-fold subsamples, the trauma-exposed sample was randomly split into ten groups of approximately equal size. The model was trained on nine-tenths of the sample (training dataset), and tested in the remaining tenth (testing dataset). This training and testing process was repeated ten times, testing on each of the groups in turn. Model-based predicted probabilities of PTSD from the ten testing datasets were used to estimate prediction performance. We next tested the internal validity of *penalized* logistic regression models using ten-fold cross-validation, to consider whether penalized models produce better prediction performance. Of note, it is not possible to test the internal validity of penalized models using the bootstrap method described above and, thus, these analyses further expand the test of sensitivity beyond different internal validation methods to also examine different regression methods. The aim of penalized regression methods – such as ridge, Least Absolute Shrinkage and Selection Operator (LASSO), and elastic net – is to address overfitting, reduce the variability of the regression estimates, and therefore improve prediction accuracy. While standard logistic regression methods obtain models that maximize a likelihood function, penalized logistic regression methods obtain models that maximize a likelihood function which also includes a penalty term. The ridge penalty is the sum of squared coefficients; the LASSO penalty is the sum of absolute coefficients; and the elastic net penalty is a combination of the ridge and LASSO penalties. LASSO and elastic net methods also allow automatic variable selection, as the coefficients of weaker predictors are shrunk towards zero. Within each training dataset, to identify the optimal penalty, we performed ten-fold cross-validation. We then used this penalty when training the model on the training dataset, and tested this model in the testing dataset. As before, this training and testing process was repeated ten times, testing on each of the testing datasets in turn; and model-based predicted probabilities of PTSD from the ten testing datasets were used to estimate prediction performance. We undertook this process for ridge, LASSO, and elastic net penalized methods.[6,7] Analyses were conducted using R 3.4.2[2] including the 'glmnet' package.[8]

PTSD risk prediction performance measures

Within these internal validations, model prediction performance was measured in terms of discrimination, calibration, and overall prediction performance:[7,9]

Discrimination is the ability of the model to distinguish between a trauma-exposed participant without PTSD and a participant with PTSD. Discrimination was assessed with area under the receiver operating characteristic curve (AUC) analysis. The AUC quantifies discrimination, and indicates the probability that a participant with PTSD has a higher model-based predicted probability of PTSD than a trauma-exposed participant without PTSD, ranging from 0·5 (no discrimination) to 1 (perfect discrimination).

Calibration is the degree of agreement between observed and model-based predicted probabilities of PTSD. Calibration was assessed with calibration-in-the-large and calibration slope. Calibration-in-the-large is the intercept of the calibration plot, and compares mean observed and predicted probabilities, indicating the extent to which predictions are systematically low or high; good calibration is demonstrated by values close to zero. Calibration slope is the regression slope with the linear predictor as the sole predictor; good calibration is demonstrated by values close to one.

Overall prediction performance captures aspects of discrimination and calibration. Overall prediction performance was assessed with the Brier score. The Brier score indicates the mean squared difference between the observed and predicted probabilities of PTSD; good prediction is demonstrated by low values. As the Brier score varies depending on the prevalence of the outcome, Brier scores scaled by their maximum possible scores were calculated, where $Brier_{scaled} = 1 - Brier / Brier_{max}$. The scaled Brier score corresponds to the proportion of the mean squared difference between observed and predicted values associated with a non-informative model that is accounted for by the current model, and ranges from 0% (non-informative prediction) to 100% (perfect prediction). Of note, it is not possible to calculate scaled Brier scores using the bootstrap internal validation method described above and, thus, scaled Brier scores were only calculated for models internally validated with cross-validation.

PTSD risk prediction sensitivity analysis to assess for bias in using twin data

We sought to examine whether the presence of non-independent observations within our twin sample may have biased our measures of prediction performance. Specifically, higher levels of similarity in the risk profiles of twins within each pair (compared to unrelated individuals) may have overestimated prediction performance if, during internal validation, the model was 'trained' on one twin and 'tested' on their co-twin. To address these concerns, we performed a sensitivity analysis using ten sub-samples consisting of only one twin per twin pair (randomly selected in twin pairs where both were trauma-exposed), and compared the average model prediction performance obtained from bootstrap internal validation in these sub-samples to the performance in the full trauma-exposed sample.

PTSD risk prediction using cumulative risk score

A risk calculator with multiple predictors may not be straight forward to use in clinical practice. Therefore, we also derived a PTSD risk calculator with a single cumulative risk score predictor that could be very easily applied in clinical settings. We generated this risk score by combining statistically significant risk factors for lifetime PTSD in trauma-exposed participants (from Table 2) into a cumulative risk score (range 0-2; using criteria: childhood victimization=1, and direct interpersonal index trauma=1). We used this variable to develop a logistic regression risk prediction model. We tested the validity of this model using 1,000 bootstrap resamples to obtain overfitting (optimism) bias-corrected estimates of prediction performance. These estimates may be over-optimistic and this method is not true internal validation because the variables used to generate the score were pre-selected based on results from this dataset. Model prediction performance was measured in terms of discrimination, calibration, and overall prediction performance (as above).

SUPPLEMENTARY RESULTS

PTSD risk prediction internal validation

The results of the bootstrap internal validation of the standard (non-penalized) logistic regression model are described in the main text, and are outlined in Supplementary Table S8. We found similar prediction performance results for internal validation of the standard (non-penalized) logistic regression model using ten-fold cross-validation (Supplementary Table S8). Therefore, we have presented only the results from the preferred bootstrap internal validation method in the main text. Additionally, we found similar prediction performance results for internal validation of the penalized logistic regression models (Supplementary Table S8), indicating that these methods do not substantially improve accuracy of our model. Therefore, we have presented the results from the standard (non-penalized) logistic regression model in the main text.

PTSD risk prediction sensitivity analysis to assess for bias in using twin data

We performed a sensitivity analysis to test whether using non-independent (twin) data biased our internal validation results. We compared bootstrap internal validation results from ten sub-samples consisting of only one twin per twin pair (randomly selected in twin pairs where both were trauma-exposed) with results from the full trauma-exposed sample, and found that the average prediction performance in the sub-samples was similar to the results in the full trauma-exposed sample. These findings indicate that using twins has not biased our results. A full description of these analyses is provided in Supplementary Table S9.

PTSD risk calculator formula

Standard logistic regression model:

Odds of lifetime PTSD in trauma-exposed young people = exp(-2·290143 + 0·410078*female sex + 0·2426267*minority ethnicity + -0·1516597*standardized child IQ + 0·0398603*standardized child internalizing symptoms + -0·0787415*standardized child externalizing symptoms + 0·4436153*child psychotic symptoms + 0·8537997*child victimization + 0·1746929*child accident + 0·3622164*socioeconomic disadvantage + 0·0018585*less than two biological parents at home + -0·2306189*family history of mental illness + 1·827269*direct interpersonal index trauma)

Predicted probability of lifetime PTSD in trauma-exposed young people = odds / (1 + odds)

A full description of these childhood characteristics is provided in Supplementary Table S1, Panel C.

PTSD risk prediction using cumulative risk score

We tested the validity of a PTSD risk calculator based on the PTSD cumulative risk score in trauma-exposed participants (see Supplementary Methods). First, higher risk scores were more common in participants with PTSD than in trauma-exposed participants without PTSD (Supplementary Table S10, Panel A), and bootstrap validated AUC was 0·73, indicating adequate discrimination of trauma-exposed participants with and without PTSD. Second, validated calibration-in-the-large was 0·00 and calibration slope was 0·99, indicating good calibration. The observed prevalences of PTSD for each risk score agreed well with bias-corrected predictions, further indicating good calibration (Supplementary Table S10, Panels B and C). Finally, this prediction model had a validated Brier score of 0·16, indicating adequate overall risk prediction performance.

SUPPLEMENTARY TABLES

Table S1. Assessment of trauma-exposure, PTSD diagnosis, clinical features, and childhood risk factors

Measure	Informant	Age, years	Description	Reporting period	Reference
**Panel A: Trauma exposure and PTSD diagnosis**					
Trauma exposure	Participant	18	Measured using structured interviews to ascertain DSM-5 diagnostic criteria (PTSD criterion A). According to these criteria, traumas are defined as events that involve exposure to actual or threatened death, serious injury, or sexual violation; which can be either directly experienced, witnessed, learned about happening to a close member of the person's social network, or experienced by enduring repeated or extreme first-hand exposure to details of the trauma. (Number of study members who participated at age-18 follow-up who had missing data = 2/2,066)	Lifetime	10
Index trauma type and category	Participant	18	Index trauma type and category were classified based on qualitative data from the PTSD interview[10] and Juvenile Victimization Questionnaire 2nd Revision (JVQ-R2)[11] adapted as a clinical interview.[12] First, we coded index traumas in a set of *trauma types*. Where index traumas consisted of several trauma types, for example both sexual and physical assault, these were all coded; therefore, trauma types were not mutually exclusive. Coded trauma types were adapted from the Composite International Diagnostic Interview (CIDI).[13] Next, we coded index traumas in a set of *trauma categories*. This variable described mutually exclusive subgroups of trauma-exposed individuals, required for analyses which compared subgroups of trauma-exposed participants. Coded trauma categories were developed based on previous research findings that interpersonal index traumas are associated with higher rates of PTSD than non-interpersonal index traumas, and that direct index traumas are associated with higher rates of PTSD than witnessed index traumas.[14-17] In order to compare these groups, we developed categories to describe whether the index trauma was interpersonal (actions of another person intentionally causing or threatening death, injury, or sexual violation), or not interpersonal (accident or illness); and within these groups further specified whether the index trauma was directly experienced by the participant, or witnessed only. Other trauma categories were network trauma (a traumatic event affecting someone in the participant's network that they learned details of, but did not did not directly experience or witness), other trauma (any other trauma that did not fall into these categories), and declined to answer (where participants declined to disclose details of their experience, often due to distress; classed as missing in analyses of predictors of PTSD (n=6)). Trauma types and categories are listed in Supplementary Table S3. Trauma dossiers from each trauma-exposed participant were independently coded by two psychiatrists blind to any other information about participants, to indicate types (inter-rater reliability of kappa=0·83) and categories (inter-rater reliability of kappa=0·89) of index traumas experienced.	Lifetime (PTSD interview); since age 12 years (JVQ-R2)	10,11
PTSD	Participant	18	Measured using structured interviews to ascertain DSM-5 diagnostic criteria. (Number of study members who participated at age-18 follow-up who had missing data = 3/2,066)	Since age 12 years, 12 months	10,18
**Panel B: Clinical features**					
Major depressive episode	Participant	18	Measured using the Diagnostic Interview Schedule for DSM-IV. (Number of study members who participated at age-18 follow-up who had missing data = 3/2,066)	12 months	19
Generalized anxiety disorder	Participant	18	Measured using the Diagnostic Interview Schedule for DSM-IV. (Number of study members who participated at age-18 follow-up who had missing data = 6/2,066)	12 months	19
Psychotic symptoms	Participant	18	Measured using structured interviews to assess for seven psychotic symptoms, validated by experts. Participants who had experienced one or more of these symptoms were classed as having experienced psychotic symptoms (binary variable). (Number of study members who participated at age-18 follow-up who had missing data = 3/2,066)	12 months	20,21
ADHD	Participant	18	Measured using structured interviews to ascertain DSM-5 diagnostic criteria. (Number of study members who participated at age-18 follow-up who had missing data = 5/2,066)	12 months	10,22
Conduct disorder	Participant	18	Measured using self-completed computer-based surveys to ascertain DSM-IV diagnostic criteria. (Number of study members who participated at age-18 follow-up who had missing data = 13/2,066)	12 months	23
Alcohol dependence	Participant	18	Measured using the Diagnostic Interview Schedule for DSM-IV. (Number of study members who participated at age-18 follow-up who had missing data = 3/2,066)	12 months	19
Cannabis dependence	Participant	18	Measured using the Diagnostic Interview Schedule for DSM-IV. (Number of study members who participated at age-18 follow-up who had missing data = 0/2,066)	12 months	19

Table S1 cont.

Measure	Informant	Age, years	Description	Reporting period	Reference
**Panel B cont.**					
Other drug dependence	Participant	18	Measured using the Diagnostic Interview Schedule for DSM-IV.<br>(Number of study members who participated at age-18 follow-up who had missing data = 0/2,066)	12 months	19
Nicotine dependence	Participant	18	Measured using the Fagerström Test of Nicotine Dependence (FTND). Nicotine dependence was defined as a FTND score ≥4.<br>(Number of study members who participated at age-18 follow-up who had missing data = 4/2,066)	At time of assessment	24,25
Count of mental health conditions	Participant	18	Count of number of the following conditions experienced: Major depressive episode, generalized anxiety disorder, psychotic symptoms, ADHD, conduct disorder, alcohol dependence, cannabis dependence, other drug dependence, and nicotine dependence (as above). Variable was coded to give values of 0, 1, 2, 3, and 4+ mental health conditions.<br>(Number of study members who participated at age-18 follow-up who had missing data = 14/2,066)	12 months	
Self-harm	Participant	18	Participants were asked if they had tried to hurt themselves to cope with stress or emotional pain.<br>(Number of study members who participated at age-18 follow-up who had missing data = 2/2,066)	Since age 12 years	26
Suicide attempt	Participant	18	Participants were asked if they had tried to kill themselves.<br>(Number of study members who participated at age-18 follow-up who had missing data = 3/2,066)	Since age 12 years	26
Violent offence	UK Police National Computer	18	UK Police National Computer records were searched for E-Risk cohort members by the UK Ministry of Justice in February 2016. The PNC includes complete history of cautions and convictions after age 10 years for individuals cautioned or convicted in the UK. Variable indicates participants who had been cautioned or convicted for a violent offence.<br>(Number of study members who participated at age-18 follow-up who had missing data = 6/2,066)	Since age 10 years	
NEET	Participant	18	Participants were asked if they were studying, working in paid employment, or pursuing a vocational qualification or apprenticeship training. Participants who were not were classed as NEET.<br>(Number of study members who participated at age-18 follow-up who had missing data = 0/2,066)	At time of assessment	27
Social isolation	Participant	18	Measured using the Multidimensional Scale of Perceived Social Support (MPSS), which assesses individuals' access to supportive relationships with family and friends. After reversing the scores so that higher scores reflected greater social isolation, participants with summed scores in the top quartile were classed as experiencing social isolation.<br>(Number of study members who participated at age-18 follow-up who had missing data = 5/2,066)	At time of assessment	28,29
Loneliness	Participant	18	Measured using four items from the UCLA Loneliness Scale (Version 3). Participants with summed scores in the top quartile were classed as experiencing loneliness.<br>(Number of study members who participated at age-18 follow-up who had missing data = 15/2,066)	At time of assessment	28,30
Service use	Participant	18	Participants were asked if they had used services of a general practitioner (GP); a psychologist, counsellor, or psychotherapist; or a psychiatrist, for mental health problems.<br>(Number of study members who participated at age-18 follow-up who had missing data: GP = 2/2,066; psychologist, counsellor, or psychotherapist = 1/2,066; psychiatrist = 1/2,066)	12 months	
**Panel C: Childhood characteristics**					
Sex	Mother	5	As reported by mothers.<br>(Number of study members who participated at age-18 follow-up who had missing data = 0/2,066)	At time of assessment	
Minority ethnicity	Mother	5	Participants whose mothers reported their ethnicity as Asian, black, or mixed race were classed as having minority ethnicity.<br>(Number of study members who participated at age-18 follow-up who had missing data = 0/2,066)	At time of assessment	
Child IQ	Participant	5	Measured using a short form of the Wechsler Preschool and Primary Scale of Intelligence—Revised (WPPSI–R). Using two subtests (Vocabulary and Block Design), children's IQs were prorated following procedures described by Sattler (1992). Results were standardized.<br>(Number of study members who participated at age-18 follow-up who had missing data = 14/2,066)	At time of assessment	31,32
Child internalizing symptoms	Mother, teacher	5, 7, 10, 12	Combined mother's Child Behavior Checklist (CBCL) and teacher's Teacher Report Form (TRF) ratings for withdrawn and anxious/depressed scales. Ratings were summed across all ages and standardized.<br>(Number of study members who participated at age-18 follow-up who had missing data = 4/2,066)	6 months	33,34

Table S1 cont.

Measure	Informant	Age, years	Description	Reporting period	Reference
**Panel C cont.**					
Child externalizing symptoms	Mother, teacher	5, 7, 10, 12	Combined mother's Child Behavior Checklist (CBCL) and teacher's Teacher Report Form (TRF) ratings for delinquent behavior and aggressive behavior scales. Ratings were summed across all ages and standardized. (Number of study members who participated at age-18 follow-up who had missing data = 4/2,066)	6 months	33,34
Child psychotic symptoms	Participant	12	Measured using structured interviews to assess for seven psychotic symptoms, validated by experts. Participants who had experienced one or more of these symptoms were classed as having experienced psychotic symptoms (binary variable). (Number of study members who participated at age-18 follow-up who had missing data = 59/2,066)	Lifetime	21
Child victimization	Mother	5, 7, 10, 12	Exposure to several types of victimization was assessed repeatedly when the children were 5, 7, 10, and 12 years of age, and dossiers have been compiled for each child with cumulative information about exposure to domestic violence between the mother and her partner, frequent bullying by peers, physical maltreatment by an adult, sexual abuse, emotional abuse/neglect, and physical neglect. (Number of study members who participated at age-18 follow-up who had missing data = 0/2,066)	Lifetime	12
Child accident	Mother	7, 10, 12	Parents were asked whether the participants had ever (or since last assessment) experienced a seriously harmful or frightening accident. (Number of study members who participated at age-18 follow-up who had missing data = 26/2,066)	Lifetime	
Socioeconomic disadvantage	Mother	5	Family socioeconomic status was defined through a standardized composite of parental income, education, and occupation. The three socioeconomic status indicators were highly correlated (r=0·57-0·67) and loaded significantly onto one latent factor. The population-wide distribution of the resulting factor was divided in tertiles, and the lowest tertile was used to indicate socioeconomic disadvantage in these analyses. (Number of study members who participated at age-18 follow-up who had missing data = 0/2,066)	At time of assessment	35
<2 biological parents at home	Mother	10	Biological mother or father left the family home at some point before age 10 years. (Number of study members who participated at age-18 follow-up who had missing data = 62/2,066)	Lifetime	
Family history of mental illness	Mother	12	Mothers were asked to report on their own mental health history and the mental health history of their biological mother, biological father, biological sisters, biological brothers, as well as the twins' biological father. Mothers were asked to report if anyone on the aforementioned list experienced difficulties with substance-use problems, alcohol problems, depression, psychosis, or suicide attempts. (Number of study members who participated at age-18 follow-up who had missing data = 56/2,066)	Lifetime	36

PTSD = post-traumatic stress disorder; ADHD = attention-deficit/hyperactivity disorder; NEET = not in education, employment, or training; IQ = intelligence quotient.

Table S2. Characteristics of the sample

	mean	(sd)
Age	18.4	0.36
Child IQ	95.9	(14.6)
Child internalizing symptoms	46.2	(26.0)
Child externalizing symptoms	65.9	(47.8)
	n	(%)
Monozygotic	1,166	(56.4)
Female sex	1,085	(52.2)
Minority ethnicity	132	(6.4)
Child psychotic symptoms	118	(5.9)
Child victimization	558	(27.0)
Child accident	630	(30.9)
<2 biological parents at home	961	(48.0)
Family history of mental illness	1,676	(83.4)

The table presents summary statistics for characteristics of the E-Risk Study members who participated in the age-18 assessments (n=2,066). Where data was missing, we have used pairwise deletion. A full description of these characteristics is provided in Supplementary Table S1, Panel C.
In addition to this information, the prevalence of mental health conditions, risk events, functional impairment, and service use in E-Risk participants at age 18 is provided in Table 1.
IQ = intelligence quotient.

Table S3. Prevalence of trauma exposure, PTSD, and PTSD burden, by index trauma type and category

	Panel A<br>Trauma exposure<br>(n=2,064)		Panel B<br>PTSD<br>in trauma-exposed<br>(n=Panel A)		Panel C<br>PTSD burden<br>(n=160)	
Any trauma	642	(31·1%)	160	(24·96%)	160	(100·0%)
**Index trauma type**[a]						
Sexual assault	54	(2·6%)	40	(74·1%)	40	(25·0%)
Physical assault	59	(2·9%)	36	(61·0%)	36	(22·5%)
Interpersonal threat	49	(2·4%)	21	(42·9%)	21	(13·1%)
Motor vehicle accident	54	(2·6%)	7	(13·0%)	7	(4·4%)
Other accident	45	(2·2%)	3	(6·7%)	3	(1·9%)
Illness	23	(1·1%)	6	(26·1%)	6	(3·8%)
Witnessed domestic violence	35	(1·7%)	17	(48·6%)	17	(10·6%)
Witnessed other interpersonal assault or threat	61	(3·0%)	8	(13·1%)	8	(5·0%)
Witnessed accident or illness	109	(5·3%)	21	(19·3%)	21	(13·1%)
Network death	95	(4·6%)	19	(20·0%)	19	(11·9%)
Other network trauma	84	(4·1%)	9	(10·7%)	9	(5·6%)
Other trauma	8	(0·4%)	0	(0·0%)	0	(0·0%)
Declined to answer	6	(0·3%)	4	(80·0%)	4	(2·5%)
**Index trauma category**[b]						
Direct (+/- witnessed) interpersonal assault or threat	138	(6·7%)	79	(57·2%)	79	(49·4%)
Direct (+/- witnessed) accident or illness	122	(5·9%)	16	(13·1%)	16	(10·0%)
Witnessed (not direct) interpersonal assault or threat	80	(3·9%)	12	(15·0%)	12	(7·5%)
Witnessed (not direct) accident or illness	109	(5·3%)	21	(19·3%)	21	(13·1%)
Network trauma	179	(8·7%)	28	(15·6%)	28	(17·5%)
Other trauma	8	(0·4%)	0	(0·0%)	0	(0·0%)
Declined to answer	6	(0·3%)	4	(80·0%)	4	(2·5%)

Panel A lists the prevalence of lifetime trauma exposure, by index trauma type and category, in the overall sample. Panel B lists the prevalence of lifetime PTSD in participants exposed to each index trauma type and category. Panel C lists the PTSD burden of each index trauma type and category, that is the percentage of all lifetime PTSD cases associated with each index trauma type and category. Where data was missing, we have used pairwise deletion. A full description of these trauma types and categories is provided in Supplementary Table S1, Panel A.

[a] Type of index trauma, not mutually exclusive – each trauma-exposed participant's index traumatic experience may consist of several trauma types, for example both sexual and physical assault.

[b] Category of index trauma, mutually exclusive – each trauma-exposed participant's index trauma is classified into one of these categories.

Table S4. Risk of clinical features

	Panel A: Trauma-exposed vs. unexposed		Panel B: PTSD vs. no PTSD		Panel C: PTSD vs. no PTSD in trauma-exposed	
	OR	(95% CI)	OR	(95% CI)	OR	(95% CI)
**Mental health conditions (past 12 months)**						
Major depressive episode	**2·16**	**(1·73, 2·71)**	**5·84**	**(4·21, 8·10)**	**4·66**	**(3·22, 6·76)**
Generalized anxiety disorder	**2·21**	**(1·58, 3·10)**	**4·83**	**(3·17, 7·35)**	**3·84**	**(2·36, 6·24)**
Psychotic symptoms	**2·64**	**(1·38, 5·04)**	**8·44**	**(4·41, 16·15)**	**9·83**	**(3·72, 25·97)**
ADHD	**1·92**	**(1·41, 2·63)**	**2·36**	**(1·52, 3·67)**	**1·67**	**(1·00, 2·78)**
Conduct disorder	**2·29**	**(1·78, 2·94)**	**2·28**	**(1·59, 3·29)**	1·37	(0·92, 2·03)
Alcohol dependence	**1·48**	**(1·12, 1·95)**	**2·62**	**(1·80, 3·81)**	**2·41**	**(1·56, 3·73)**
Cannabis dependence	**2·25**	**(1·43, 3·54)**	**2·34**	**(1·29, 4·22)**	1·44	(0·75, 2·77)
Other drug dependence	**3·52**	**(1·36, 9·12)**	**7·87**	**(3·00, 20·62)**	**5·46**	**(1·58, 18·84)**
Nicotine dependence	**1·75**	**(1·27, 2·42)**	**3·21**	**(2·14, 4·84)**	**2·74**	**(1·72, 4·38)**
Any of above	**1·93**	**(1·59, 2·34)**	**4·79**	**(3·28, 6·98)**	**3·66**	**(2·45, 5·48)**
**Risk events (since age 10-12 years)**						
Self-harm	**3·61**	**(2·77, 4·70)**	**8·05**	**(5·64, 11·48)**	**4·69**	**(3·16, 6·95)**
Suicide attempt	**4·85**	**(2·89, 8·13)**	**10·17**	**(6·21, 16·65)**	**5·80**	**(3·18, 10·56)**
Violent offence	**1·68**	**(1·17, 2·40)**	**3·08**	**(1·82, 5·23)**	**2·70**	**(1·43, 5·09)**
**Functional impairment (at time of assessment)**						
NEET	**2·09**	**(1·58, 2·76)**	**3·24**	**(2·17, 4·84)**	**2·31**	**(1·47, 3·63)**
Social isolation	**1·50**	**(1·22, 1·84)**	**2·75**	**(1·97, 3·84)**	**2·44**	**(1·69, 3·55)**
Loneliness	**1·82**	**(1·47, 2·24)**	**3·42**	**(2·42, 4·84)**	**2·70**	**(1·84, 3·96)**
**Service use for mental health (past 12 months)**						
General practitioner	**2·19**	**(1·64, 2·93)**	**4·48**	**(3·02, 6·65)**	**3·45**	**(2·17, 5·49)**
Psychologist, psychotherapist, or counsellor	**1·90**	**(1·32, 2·75)**	**4·07**	**(2·60, 6·38)**	**3·60**	**(2·10, 6·16)**
Psychiatrist	**2·59**	**(1·41, 4·77)**	**7·18**	**(3·80, 13·57)**	**6·57**	**(2·80, 15·41)**

The table presents unadjusted odds ratios (95% confidence intervals) for the associations between lifetime trauma exposure and each clinical feature in the overall sample (Panel A), between lifetime PTSD and each clinical feature in the overall sample (Panel B), and between lifetime PTSD and each clinical feature in trauma-exposed participants (Panel C), all calculated using logistic regression models with robust standard errors accounting for clustering within families. Where data was missing, we have used pairwise deletion. A full description of these clinical features is provided in Supplementary Table S1, Panel B.
Bold text signifies statistical significance with p<0·05.
PTSD = post-traumatic stress disorder; ADHD = attention-deficit/hyperactivity disorder; NEET = not in education, employment, or training.

Table S5. Prevalence of clinical features (12-month PTSD)

	Panel A: Overall sample (n=2,066)		Panel B: Trauma-unexposed (n=1,422)		Panel C: Trauma-exposed (n=642)		Panel D: Overall sample, no 12-month PTSD (n=1,973)		Panel E: Trauma-exposed, no 12-month PTSD (n=551)		Panel F: 12-month PTSD (n=90)	
	n	(%)	n	(%)	n	(%)	n	(%)	n	(%)	n	(%)
**Mental health conditions (past 12 months)**												
Major depressive episode	414	(20·1)	227	(16·0)	187	(29·2)	361	(18·3)	134	(24·4)	52	(57·8)
Generalized anxiety disorder	153	(7·4)	79	(5·6)	74	(11·5)	124	(6·3)	45	(8·2)	29	(32·2)
Psychotic symptoms	39	(1·9)	18	(1·3)	21	(3·3)	29	(1·5)	11	(2·0)	9	(10·0)
ADHD	171	(8·3)	94	(6·6)	77	(12·0)	152	(7·7)	58	(10·5)	18	(20·0)
Conduct disorder	309	(15·1)	162	(11·5)	146	(22·9)	282	(14·4)	120	(21·9)	25	(28·1)
Alcohol dependence	263	(12·7)	161	(11·3)	102	(15·9)	241	(12·2)	80	(14·5)	21	(23·3)
Cannabis dependence	89	(4·3)	45	(3·2)	44	(6·9)	78	(4·0)	33	(6·0)	11	(12·2)
Other drug dependence	18	(0·9)	7	(0·5)	11	(1·7)	14	(0·7)	7	(1·3)	4	(4·4)
Nicotine dependence	183	(8·9)	104	(7·3)	78	(12·2)	158	(8·0)	54	(9·8)	23	(25·6)
Any of above	886	(43·5)	538	(38·4)	346	(54·6)	812	(41·7)	274	(50·4)	71	(79·8)
**Risk events (since age 10-12 years)**												
Self-harm	280	(13·6)	120	(8·4)	160	(25·0)	235	(11·9)	115	(20·9)	44	(48·9)
Suicide attempt	79	(3·8)	26	(1·8)	53	(8·3)	60	(3·0)	34	(6·2)	18	(20·0)
Violent offence	99	(4·8)	57	(4·0)	42	(6·6)	89	(4·5)	32	(5·8)	10	(11·2)
**Functional impairment (at time of assessment)**												
NEET	239	(11·6)	128	(9·0)	110	(17·1)	212	(10·7)	84	(15·2)	25	(27·8)
Social isolation	577	(28·0)	360	(25·4)	217	(33·8)	530	(26·9)	170	(30·9)	46	(51·1)
Loneliness	541	(26·4)	320	(22·6)	221	(34·7)	489	(25·0)	169	(31·0)	51	(57·3)
**Service use for mental health (past 12 months)**												
General practitioner	215	(10·4)	113	(8·0)	102	(15·9)	182	(9·2)	69	(12·5)	32	(35·6)
Psychologist, psychotherapist, or counsellor	133	(6·4)	73	(5·1)	60	(9·3)	112	(5·7)	39	(7·1)	20	(22·2)
Psychiatrist	45	(2·2)	21	(1·5)	24	(3·7)	31	(1·6)	10	(1·8)	14	(15·6)

The table lists the prevalence of each clinical feature in the overall sample (Panel A), trauma-unexposed participants (Panel B), trauma-exposed participants (Panel C), participants without 12-month PTSD in the overall sample (Panel D), participants without 12-month PTSD in the trauma-exposed sub-sample (Panel E), and participants with 12-month PTSD (Panel F)· Where data was missing, we have used pairwise deletion. A full description of these clinical features is provided in Supplementary Table S1, Panel B.
PTSD = post-traumatic stress disorder; ADHD = attention-deficit/hyperactivity disorder; NEET = not in education, employment, or training.

Table S6. Risk of clinical features (12-month PTSD)

	Panel A: Trauma-exposed vs. unexposed		Panel B: 12-month PTSD vs. no 12-month PTSD		Panel C: 12-month PTSD vs. no 12-month PTSD in trauma-exposed	
	OR	(95% CI)	OR	(95% CI)	OR	(95% CI)
**Mental health conditions (past 12 months)**						
Major depressive episode	**2·16**	**(1·73, 2·71)**	**6·10**	**(4·01, 9·27)**	**4·25**	**(2·72, 6·62)**
Generalized anxiety disorder	**2·21**	**(1·58, 3·10)**	**7·07**	**(4·42, 11·29)**	**5·34**	**(3·18, 8·95)**
Psychotic symptoms	**2·64**	**(1·38, 5·04)**	**7·44**	**(3·48, 15·90)**	**5·44**	**(2·29, 12·95)**
ADHD	**1·92**	**(1·41, 2·63)**	**2·99**	**(1·76, 5·06)**	**2·12**	**(1·19, 3·77)**
Conduct disorder	**2·29**	**(1·78, 2·94)**	**2·33**	**(1·47, 3·68)**	1·39	(0·86, 2·25)
Alcohol dependence	**1·48**	**(1·12, 1·95)**	**2·18**	**(1·31, 3·63)**	**1·79**	**(1·03, 3·10)**
Cannabis dependence	**2·25**	**(1·43, 3·54)**	**3·38**	**(1·72, 6·67)**	**2·19**	**(1·07, 4·47)**
Other drug dependence	**3·52**	**(1·36, 9·12)**	**6·51**	**(2·09, 20·31)**	**3·61**	**(1·03, 12·69)**
Nicotine dependence	**1·75**	**(1·27, 2·42)**	**3·93**	**(2·39, 6·48)**	**3·15**	**(1·84, 5·40)**
Any of above	**1·93**	**(1·59, 2·34)**	**5·51**	**(3·27, 9·28)**	**3·89**	**(2·27, 6·65)**
**Risk events (since age 10-12 years)**						
Self-harm	**3·61**	**(2·77, 4·70)**	**7·07**	**(4·54, 11·02)**	**3·62**	**(2·28, 5·75)**
Suicide attempt	**4·85**	**(2·89, 8·13)**	**7·96**	**(4·50, 14·09)**	**3·79**	**(2·04, 7·04)**
Violent offence	**1·68**	**(1·17, 2·40)**	**2·67**	**(1·32, 5·40)**	2·05	(0·96, 4·39)
**Functional impairment (at time of assessment)**						
NEET	**2·09**	**(1·58, 2·76)**	**3·19**	**(1·97, 5·18)**	**2·14**	**(1·28, 3·57)**
Social isolation	**1·50**	**(1·22, 1·84)**	**2·84**	**(1·81, 4·45)**	**2·34**	**(1·47, 3·74)**
Loneliness	**1·82**	**(1·47, 2·24)**	**4·03**	**(2·54, 6·41)**	**2·99**	**(1·85, 4·86)**
**Service use for mental health (past 12 months)**						
General practitioner	**2·19**	**(1·64, 2·93)**	**5·42**	**(3·39, 8·67)**	**3·85**	**(2·31, 6·41)**
Psychologist, psychotherapist, or counsellor	**1·90**	**(1·32, 2·75)**	**4·74**	**(2·83, 7·96)**	**3·75**	**(2·12, 6·64)**
Psychiatrist	**2·59**	**(1·41, 4·77)**	**11·53**	**(5·87, 22·65)**	**9·97**	**(4·33, 22·95)**

The table presents unadjusted odds ratios (95% confidence intervals) for the associations between lifetime trauma exposure and each clinical feature in the overall sample (Panel A), between 12-month PTSD and each clinical feature in the overall sample (Panel B), and between 12-month PTSD and each clinical feature in trauma-exposed participants (Panel C), all calculated using logistic regression models with robust standard errors accounting for clustering within families. Where data was missing, we have used pairwise deletion. A full description of these clinical features is provided in Supplementary Table S1, Panel B.
Bold text signifies statistical significance with p<0·05.
PTSD = post-traumatic stress disorder; ADHD = attention-deficit/hyperactivity disorder; NEET = not in education, employment, or training.

Table S7. Risk of clinical features, adjusted for other mental health conditions

		Trauma-exposed vs. unexposed		PTSD vs. no PTSD		PTSD vs. no PTSD in trauma-exposed	
		OR	(95% CI)	OR	(95% CI)	OR	(95% CI)
**Adjusted for major depressive episode**	**Risk events (since age 10-12 years)**						
	Self-harm	**3·03**	**(2·29, 4·01)**	**4·93**	**(3·26, 7·45)**	**3·12**	**(2·02, 4·81)**
	Suicide attempt	**3·66**	**(2·17, 6·18)**	**5·55**	**(3·21, 9·59)**	**3·53**	**(1·82, 6·83)**
	Violent offence	**1·53**	**(1·06, 2·21)**	**2·58**	**(1·45, 4·58)**	**2·30**	**(1·15, 4·60)**
	**Functional impairment (at time of assessment)**						
	NEET	**1·86**	**(1·41, 2·47)**	**2·41**	**(1·57, 3·70)**	**1·75**	**(1·07, 2·87)**
	Social isolation	**1·35**	**(1·09, 1·66)**	**2·11**	**(1·50, 2·98)**	**1·86**	**(1·26, 2·74)**
	Loneliness	**1·53**	**(1·23, 1·91)**	**2·13**	**(1·48, 3·07)**	**1·68**	**(1·11, 2·54)**
	**Service use for mental health (past 12 months)**						
	General practitioner	**1·63**	**(1·19, 2·23)**	**2·20**	**(1·37, 3·53)**	**2·04**	**(1·20, 3·48)**
	Psychologist, psychotherapist, or counsellor	1·31	(0·88, 1·95)	**1·81**	**(1·09, 3·01)**	**1·91**	**(1·06, 3·44)**
	Psychiatrist	1·80	(0·98, 3·28)	**3·41**	**(1·76, 6·60)**	**3·14**	**(1·28, 7·73)**
**Adjusted for psychotic symptoms**	**Risk events (since age 10-12 years)**						
	Self-harm	**3·52**	**(2·68, 4·61)**	**7·29**	**(5·04, 10·53)**	**4·16**	**(2·77, 6·23)**
	Suicide attempt	**4·52**	**(2·66, 7·67)**	**8·60**	**(5·01, 14·75)**	**5·44**	**(2·93, 10·10)**
	Violent offence	**1·64**	**(1·15, 2·36)**	**2·95**	**(1·69, 5·15)**	**2·70**	**(1·42, 5·15)**
	**Functional impairment (at time of assessment)**						
	NEET	**2·03**	**(1·54, 2·69)**	**3·01**	**(2·00, 4·52)**	**2·07**	**(1·31, 3·29)**
	Social isolation	**1·47**	**(1·20, 1·81)**	**2·64**	**(1·88, 3·71)**	**2·37**	**(1·62, 3·46)**
	Loneliness	**1·77**	**(1·44, 2·19)**	**3·17**	**(2·23, 4·52)**	**2·42**	**(1·63, 3·58)**
	**Service use for mental health (past 12 months)**						
	General practitioner	**2·10**	**(1·56, 2·82)**	**3·97**	**(2·61, 6·03)**	**3·21**	**(1·99, 5·17)**
	Psychologist, psychotherapist, or counsellor	**1·74**	**(1·19, 2·55)**	**3·20**	**(1·92, 5·35)**	**3·04**	**(1·70, 5·41)**
	Psychiatrist	**2·25**	**(1·18, 4·27)**	**5·11**	**(2·33, 11·23)**	**5·24**	**(1·99, 13·75)**
**Adjusted for count of mental health conditions**	**Risk events (since age 10-12 years)**						
	Self-harm	**2·68**	**(2·01, 3·57)**	**4·69**	**(3·10, 7·11)**	**3·19**	**(2·06, 4·94)**
	Suicide attempt	**3·09**	**(1·81, 5·29)**	**4·82**	**(2·68, 8·69)**	**3·59**	**(1·81, 7·12)**
	Violent offence	1·18	(0·79, 1·77)	1·69	(0·92, 3·09)	1·83	(0·90, 3·70)
	**Functional impairment (at time of assessment)**						
	NEET	**1·62**	**(1·19, 2·19)**	**1·85**	**(1·16, 2·95)**	1·50	(0·90, 2·52)
	Social isolation	**1·27**	**(1·03, 1·57)**	**2·05**	**(1·44, 2·92)**	**1·91**	**(1·29, 2·82)**
	Loneliness	**1·41**	**(1·13, 1·76)**	**1·99**	**(1·37, 2·91)**	**1·62**	**(1·05, 2·51)**
	**Service use for mental health (past 12 months)**						
	General practitioner	**1·54**	**(1·12, 2·13)**	**2·33**	**(1·43, 3·82)**	**2·26**	**(1·32, 3·88)**
	Psychologist, psychotherapist, or counsellor	1·26	(0·83, 1·89)	**1·95**	**(1·15, 3·32)**	**2·16**	**(1·19, 3·92)**
	Psychiatrist	1·60	(0·86, 2·99)	**3·29**	**(1·62, 6·69)**	**3·59**	**(1·41, 9·14)**

The table lists odds ratios (95% confidence intervals) for the associations between [1] lifetime trauma exposure and each clinical feature in the overall sample, [2] lifetime PTSD and each clinical feature in the overall sample, and [3] lifetime PTSD and each clinical feature in trauma-exposed participants, adjusted for mental health conditions as indicated in the first column (major depressive episode, psychotic symptoms, or count of mental health conditions), calculated using logistic regression models with robust standard errors accounting for clustering within families. Where data was missing, we have used pairwise deletion. A full description of these clinical features is provided in Supplementary Table S1, Panel B.

Bold text signifies statistical significance with $p<0·05$. PTSD = post-traumatic stress disorder; NEET = not in education, employment, or training.

Table S8. PTSD risk calculator sensitivity analysis to compare different methods of internal validation and penalized models

		Logistic regression model, bootstrap internal validation	Logistic regression model, ten-fold cross-validation internal validation	Ridge penalized logistic regression model, ten-fold cross-validation internal validation	LASSO penalized logistic regression model, ten-fold cross-validation internal validation	Elastic net penalized logistic regression model, ten-fold cross-validation internal validation
**Risk prediction performance measure**	**Discrimination**					
	AUC	0·74	0·74	0·74	0·74	0·74
	**Calibration**					
	Calibration-in-the-large	-0·10	0·00	0·00	0·00	0·00
	Calibration slope	0·90	0·87	1·05	1·03	1·05
	**Overall**					
	Brier	0·15	0·15	0·15	0·15	0·15
	$Brier_{scaled}$	–	0.17	0.17	0.17	0.17
**Predictors, odds ratios**	**Individual characteristics**					
	Female sex	1·51	1·51	1·44	1·35	1·38
	Minority ethnicity	1·27	1·27	1·26	–	1·03
	Child IQ[a]	0·86	0·86	0·89	0·92	0·91
	Child internalizing symptoms[a]	1·04	1·04	1·06	–	–
	Child externalizing symptoms[a]	0·92	0·92	0·96	–	–
	Child psychotic symptoms	1·56	1·56	1·49	1·25	1·31
	Child victimization	2·35	2·35	1·95	2·00	1·97
	Child accident	1·19	1·19	1·13	–	–
	**Family characteristics**					
	Socioeconomic disadvantage	1·44	1·44	1·36	1·25	1·28
	<2 biological parents at home	1·00	1·00	1·03	–	–
	Family history of mental illness	0·79	0·79	0·83	–	0·96
	**Index trauma category**					
	Direct (+/- witnessed) interpersonal assault or threat	6·22	6·22	4·54	5·40	5·10

The PTSD risk calculator was first derived using the standard multivariate logistic regression model predicting lifetime PTSD in trauma-exposed participants (Table 2). The table shows the internally validated risk prediction performance of this model, using bootstrap resampling and ten-fold cross-validation. The PTSD risk calculator was then derived using penalized multivariate logistic regression models predicting lifetime PTSD in trauma-exposed participants (penalty terms were an average of the optimal penalties found across the ten folds). The table shows the internally validated risk prediction performance of these models, using ten-fold cross-validation. The table also shows odds ratios for predictors based on these models.

Table S9. PTSD risk calculator sensitivity analysis to assess for bias in using twin data

Risk prediction performance measure	Panel A: Trauma-exposed participants (n=605)	Panel B: Trauma-exposed participants, subsamples consisting of one twin per twin pair (n=484) 1	2	3	4	5	6	7	8	9	10	Average
**Discrimination**												
AUC	0·74	0·74	0·74	0·76	0·75	0·76	0·76	0·77	0·79	0·77	0·74	0·76
**Calibration**												
Calibration-in-the-large	-0·10	-0·13	-0·13	-0·12	-0·13	-0·12	-0·11	-0·11	-0·11	-0·12	-0·12	-0·12
Calibration slope	0·90	0·87	0·87	0·88	0·87	0·88	0·88	0·89	0·88	0·88	0·88	0·88
**Overall**												
Brier	0·15	0·15	0·15	0·14	0·15	0·14	0·15	0·14	0·14	0·14	0·15	0·15

The PTSD risk calculator was derived using the multivariate logistic regression model predicting lifetime PTSD in trauma-exposed participants (Table 2). Panel A shows the bootstrap internally validated risk prediction performance of this model which was developed in all trauma-exposed participants. We were concerned that using non-independent (twin) data may bias our internal validation results, and undertook a sensitivity analysis to test this, shown in Panel B. In this sensitivity analysis, we tested ten subsamples consisting of only one twin per twin pair (randomly selected in twin pairs where both were trauma-exposed). The average prediction performance was similar to the results of the full trauma-exposed sample (Panel A), indicating that using twins has not biased our results.

Table S10. PTSD cumulative risk score in trauma-exposed participants

Risk score	Panel A: Trauma-exposed, no PTSD (n=456) n	(%)	Panel A: Trauma-exposed, PTSD (n=149) n	(%)	Panel B: Observed PTSD prevalence (n=Panel A) n	(%)	Panel B: Observed PTSD odds ratio (n=605) OR	(95% CI)	Panel C: Bias-corrected PTSD predicted probability
0	295	(64·7)	36	(24·2)	36	(10·9)	Reference		0·121
1	141	(30·9)	74	(49·7)	74	(34·4)	**4·30**	**(2·77, 6·67)**	0·345
2	20	(4·4)	39	(26·2)	39	(66·1)	**15·98**	**(8·40, 30·40)**	0·620

We generated this PTSD cumulative risk score by combining statistically significant risk factors for lifetime PTSD in trauma-exposed participants (from Table 2) into a cumulative risk score (range 0-2; using criteria: childhood victimization=1, and direct interpersonal index trauma=1). Panel A lists the percentage of trauma-exposed participants without PTSD and those with lifetime PTSD who had each risk score. Panel B lists the observed prevalence of lifetime PTSD in trauma-exposed participants with each risk score, and the odds ratios (95% confidence intervals) for associations between each risk score versus 0 and PTSD, calculated using logistic regression with robust standard errors. We tested the validity of this model using 1,000 bootstrap resamples to obtain overfitting (optimism) bias-corrected estimates of prediction performance. Panel C lists the bias-corrected predicted probability of lifetime PTSD in trauma-exposed participants with each risk score, based on this validation.

SUPPLEMENTARY REFERENCES

1 Harrell FE, Lee KL, Mark DB. Multivariable prognostic models: issues in developing models, evaluating assumptions and adequacy, and measuring and reducing errors. *Stat Med* 1996; **15**: 361–87.

2 R Core Team. R: a language and environment for statistical computing. Vienna: R Foundation for Statistical Computing, 2017 http://www.R-project.org/.

3 Harrell FE. rms: Regression Modeling Strategies. 2018 https://CRAN.R-project.org/package=rms.

4 Smith GCS, Seaman SR, Wood AM, Royston P, White IR. Correcting for optimistic prediction in small data sets. *Am J Epidemiol* 2014; **180**: 318–24.

5 Steyerberg EW, Harrell FE, Borsboom GJ, Eijkemans MJ, Vergouwe Y, Habbema JD. Internal validation of predictive models: efficiency of some procedures for logistic regression analysis. *J Clin Epidemiol* 2001; **54**: 774–81.

6 Hastie T, Tibshirani R, Friedman J. The elements of statistical learning: data mining, inference, and prediction, Second edition. New York: Springer, 2009.

7 Steyerberg EW. Clinical prediction models. New York: Springer-Verlag, 2009.

8 Friedman J, Hastie T, Tibshirani R. Regularization paths for generalized linear models via coordinate descent. *J Stat Softw* 2010; **33**: 1–22.

9 Steyerberg EW, Vickers AJ, Cook NR, *et al.* Assessing the performance of prediction models: a framework for traditional and novel measures. *Epidemiology* 2010; **21**: 128–38.

10 American Psychiatric Association. Diagnostic and Statistical Manual of Mental Disorders, 5th edition. Washington DC: American Psychiatric Association, 2013.

11 Finkelhor D, Hamby S, Turner H, Ormrod R. The Juvenile Victimization Questionnaire: 2nd Revision (JVQ-R2). Durham: Crimes Against Children Research Center, 2011.

12 Fisher HL, Caspi A, Moffitt TE, *et al.* Measuring adolescents' exposure to victimization: the Environmental Risk (E-Risk) Longitudinal Twin Study. *Dev Psychopathol* 2015; **27**: 1399–416.

13 Kessler RC, Üstün TB. The World Mental Health (WMH) Survey Initiative version of the World Health Organization (WHO) Composite International Diagnostic Interview (CIDI). *Int J Methods in Psychiatr Res* 2006; **13**: 93–121.

14 McLaughlin KA, Koenen KC, Hill ED, *et al.* Trauma exposure and posttraumatic stress disorder in a national sample of adolescents. *J Am Acad Child Adolesc Psychiatry* 2013; **52**: 815–830.e14.

15 Kessler RC, Sonnega A, Bromet E, Hughes M, Nelson CB. Posttraumatic stress disorder in the National Comorbidity Survey. *Arch Gen Psychiatry* 1995; **52**: 1048–60.

16 Creamer M, Burgess P, McFarlane AC. Post-traumatic stress disorder: findings from the Australian National Survey of Mental Health and Well-being. *Psychol Med* 2001; **31**: 1237–47.

17 Darves-Bornoz J-M, Alonso J, de Girolamo G, *et al.* Main traumatic events in Europe: PTSD in the European study of the epidemiology of mental disorders survey. *J Trauma Stress* 2008; **21**: 455–62.

18 Friedman MJ, Resick PA, Bryant RA, Brewin CR. Considering PTSD for DSM-5. *Depress Anxiety* 2011; **28**: 750–69.

19 Robins LN, Cottler L, Bucholz KK, Compton W. Diagnostic Interview Schedule for DSM-IV. St Louis: Washington University School of Medicine, 1995.

20 Newbury J, Arseneault L, Caspi A, Moffitt TE, Odgers CL, Fisher HL. Cumulative effects of neighborhood social adversity and personal crime victimization on adolescent psychotic experiences. *Schizophr Bull* 2018; **44**: 348–58.

21 Polanczyk G, Moffitt TE, Arseneault L, *et al.* Etiological and clinical features of childhood psychotic symptoms: results from a birth cohort. *Arch Gen Psychiatry* 2010; **67**: 328–38.

22 Agnew-Blais JC, Polanczyk GV, Danese A, Wertz J, Moffitt TE, Arseneault L. Evaluation of the persistence, remission, and emergence of attention-deficit/hyperactivity disorder in young adulthood. *JAMA Psychiatry* 2016; published online May 18. DOI:10.1001/jamapsychiatry.2016.0465.

23 American Psychiatric Association. Diagnostic and Statistical Manual of Mental Disorders, 4th edition. Washington DC: American Psychiatric Association, 1994.

24 Heatherton TF, Kozlowski LT, Frecker RC, Fagerström KO. The Fagerström Test for Nicotine Dependence: a revision of the Fagerström Tolerance Questionnaire. *Br J Addict* 1991; **86**: 1119–27.

25 Belsky DW, Moffitt TE, Baker TB, *et al.* Polygenic risk and the developmental progression to heavy, persistent smoking and nicotine dependence: evidence from a 4-decade longitudinal study. *JAMA Psychiatry* 2013; **70**: 534–42.

26 Baldwin JR, Arseneault L, Caspi A, *et al.* Adolescent Victimization and Self-Injurious Thoughts and Behaviors: A Genetically Sensitive Cohort Study. *J Am Acad Child Adolesc Psychiatry* 2018; published online Dec. DOI:10.1016/j.jaac.2018.07.903.

27 Goldman-Mellor S, Caspi A, Arseneault L, *et al.* Committed to work but vulnerable: self-perceptions and mental health in NEET 18-year olds from a contemporary British cohort. *J Child Psychol Psychiatry* 2016; **57**: 196–203.

28 Matthews T, Danese A, Wertz J, *et al.* Social isolation, loneliness and depression in young adulthood: a behavioural genetic analysis. *Soc Psychiatry Psychiatr Epidemiol* 2016; **51**: 339–48.

29 Zimet GD, Dahlem NW, Zimet SG, Farley GK. The Multidimensional Scale of Perceived Social Support. *J Pers Assess* 1988; **52**: 30–41.

30 Russell DW. UCLA Loneliness Scale (Version 3): reliability, validity, and factor structure. *J Pers Assess* 1996; **66**: 20–40.

31 Wechsler D. Wechsler preschool and primary scale of intelligence–revised. London: Psychological Corporation, 1990.

32 Sattler JM. Assessment of children: WISC-III and WPPSI-R supplement. San Diego: Jerome M Sattler, 1992.

33 Achenbach TM. Manual for the Child Behavior Checklist/4-18 and 1991 profile. Burlington: University of Vermont Department of Psychiatry, 1991.

34 Achenbach TM. Manual for Teacher's Report Form and 1991 profile. Burlington: University of Vermont Department of Psychiatry, 1991.

35 Trzesniewski KH, Moffitt TE, Caspi A, Taylor A, Maughan B. Revisiting the association between reading achievement and antisocial behavior: new evidence of an environmental explanation from a twin study. *Child Dev* 2006; **77**: 72–88.

36 Odgers CL, Caspi A, Russell MA, Sampson RJ, Arseneault L, Moffitt TE. Supportive parenting mediates neighborhood socioeconomic disparities in children's antisocial behavior from ages 5 to 12. *Dev Psychopathol* 2012; **24**: 705–21.
